# A new generalized family of distributions based on combining Marshal-Olkin transformation with T-X family

**DOI:** 10.1371/journal.pone.0263673

**Published:** 2022-02-09

**Authors:** Hadeel Klakattawi, Dawlah Alsulami, Mervat Abd Elaal, Sanku Dey, Lamya Baharith

**Affiliations:** 1 Department of Statistics, Faculty Science, King Abdul-Aziz University, Jeddah, Saudi Arabia; 2 Department of Statistics, St. Anthony’s College, Shillong, Meghalaya, India; University of Bradford, UNITED KINGDOM

## Abstract

Data analysis in real life often relies mainly on statistical probability distributions. However, data arising from different fields such as environmental, financial, biomedical sciences and other areas may not fit the classical distributions. Therefore, the need arises for developing new distributions that would capture high degree of skewness and kurtosis and enhance the goodness-of-fit in empirical distribution. In this paper, we introduce a novel family of distributions which can extend some popular classes of distributions to include different new versions of the baseline distributions. The proposed family of distributions is referred as the Marshall-Olkin Weibull generated family. The proposed family of distributions is a combination of Marshall-Olkin transformation and the Weibull generated family. Two special members of the proposed family are investigated. A variety of shapes for the densities and hazard rate are presented of the considered sub-models. Some of the main mathematical properties of this family are derived. The estimation for the parameters is obtained via the maximum likelihood method. Moreover, the performance of the estimators for the considered members is examined through simulation studies in terms of bias and root mean square error. Besides, based on the new generated family, the log Marshall-Olkin Weibull-Weibull regression model for censored data is proposed. Finally, COVID-19 data and three lifetime data sets are used to demonstrate the importance of the newly proposed family. Through such an applications, it is shown that this family of distributions provides a better fit when compared with other competitive distributions.

## 1 Introduction

Selecting a suitable statistical distribution for modeling and analyzing data is of great importance in order to achieve more accurate decisions. Over the years, many statistical distributions have been proposed to fit different shapes of the data. Applying classical distributions to fit these data sets may lead to inaccurate results. Hence, the need for modifying the standard distributions is clearly evident.

Recently, many methods have been proposed to obtain more flexible distributions that can reflect the data behavior in many situations more accurately. Adding a new parameter to existing distributions is one popular method that has attracted more attention in literature. [1] proposed the exponentiated method by raising a new parameter to the cumulative distribution function (cdf) of any distribution.

Additionally, [2], presented a new modified method for obtaining more adaptable distributions called Marshall-Olkin (MO) family of distributions. The method relies on extending a given distribution by including an additional shape parameter called a tilt parameter, [3]. To illustrate, consider a random variable *X* from a baseline distribution and such variable has a cdf *G*(*x*; ***τ***). Then, for the MO family, its cdf and its probability density function (pdf) are, respectively, given by

$$\begin{array}{c} F^{MO}\left(x\right)=\frac{G(x;\tau )}{\alpha +\bar{\alpha }\left(x;\tau \right)}, \end{array}$$ (1)

and

$$\begin{array}{c} f^{MO}\left(x\right)=\frac{\alpha g(x;\tau )}{{\left(\alpha +\bar{\alpha }G\left(x;\tau \right)\right)}^{2}}, \end{array}$$ (2)

where *α* > 0 is the shape (tilt) parameter, $\bar{\alpha }=1-\alpha$ and ***τ*** is a vector of parameters for the baseline model. The MO transformation has some attractable characteristics which can add more flexibility to any baseline distribution. This encouraged many researchers to apply the MO transformation to common classical distributions such as the Pareto distribution by [4], Weibull distribution by [5, 6], the three parameter Weibull by [7], Lomax distribution by [8], gamma distribution by [9], Uniform distribution by [10], log-logistic distribution by [11], inverted Kumaraswamy distribution by [12], inverse Lomax distribution by [13], kappa distribution by [14], generalized Pareto distribution by [15], Gumbel-Lomax distribution by [16], power Lomax distribution by [17] and inverse log-logistic distribution by [18], among others.

The MO transformation can be applied on family of distributions. Specifically, a compounding technique can be used to combine the MO family with other generated classes to obtain some new families that provide distribution with greater flexibility in modeling. This method can be defined by taking any family of distributions as the baseline cdf in Eq (1). Several families have been generalized using this method, for instance, [19] introduced the MO extended Weibull family, [20] suggested the MO Kumarswamy-G family, [21] proposed the MO odd Lindley-G family, [22] proposed the MO alpha power family of distribution, [23] considered the MO Topp Leone-G family and [24] suggested the MO odd Burr III-G family and among many others.

Another way to develop a new flexible distribution is by the transformed-transformer (T-X) family proposed by [25]. The T-X method is widely applicable because any continuous distribution can be used as a generator. In other words, for any continuous random variable *X*, the cdf and pdf for the T-X family with a vector of parameters ***ζ*** = (***θ***, ***τ***) are, respectively, defined by

$$\begin{array}{c} F\left(x;\zeta \right)=\int_{a}^{W(G(x;\tau ))}r\left(t;\theta \right)dt,\;\text{and}\;f\left(x;\zeta \right)=[\frac{d}{dx}W\left(G\left(x;\tau \right)\right)]r[W(G(x;\tau ))], \end{array}$$

where *a* is a real number, *r*(*t*; ***θ***) is the generator pdf of a random variable *T* and *W*(*G*(*x*; ***τ***)) is function of the cdf of the random variable *X*. [25, 26] presented the Weibull-G family as an example of the T-X family based on the Weibull distribution with parameters *c* and *β* as a generator. That is, for any distribution with cdf *G*(*x*; ***τ***) and vector of parameters; ***τ***, the cdf of the Weibull-G family can be expressed as follows

$$\begin{array}{cc} F^{WG}\left(x;c,\beta ,\tau \right) & =1-e^{-{\left(\frac{-\text{log}(1-G(x;\tau ))}{\beta }\right)}^{c}}, \end{array}$$ (3)

and the corresponding pdf is of the form

$$\begin{array}{c} \begin{array}{cc} f^{WG}\left(x;c,\beta ,\tau \right) & =\frac{c}{\beta }\frac{g(x;\tau )}{1-G(x;\tau )}{\left(\frac{-\text{log}(1-G(x;\tau ))}{\beta }\right)}^{c-1}e^{-{\left(\frac{-\text{log}(1-G(x;\tau ))}{\beta }\right)}^{c}}. \end{array} \end{array}$$ (4)

where *c* > 0 is the shape parameter and *β* > 0 is a scale parameter.

Many studies have been considered of some members of the Weibull-G family, such as the Weibull-Pareto by [27], the Weibull-Rayleigh by [28], the Weibull-Lomax by [29] and more lately the Weibull-gamma by [30].

Recently, attempts have been made to generalize the Weibull-G family. For example, [31] introduced a Beta Weibull-G class of distributions, [32] generalized the Weibull-G family by the combination with Kumaraswamy-G family to obtain the Kumaraswamy Weibull-G family and also the Weibull-G family was combined by [33] with the gamma-generator for defining the gamma Weibull-G, among others.

The first objective of this paper is to introduce a novel generalization for the Weibull-G family, called MO Weibull-G (MOW-G) family and derive some of its properties. The construction of this family is the combination of the MO family and the Weibull-G. Marshall-Olkin Weibull-Exponential (MOW-E) and Marshall-Olkin Weibull-Weibull (MOW-W) distributions are special cases of the MOW-G distribution. The proposed family of distributions provide better fits than some well known lifetime distributions. The importance of this new family of distributions is the ability of describing decreasing, increasing, bath-tub and upside down bath-tub shaped hazard rate functions which is extensively used in many real life data. Besides, MOW-G family is a suitable model for fitting positively skewed data which may not be adequately modelled by many other distributions. Thus, it can be used to fit data related to public health, biomedical studies, industrial reliability, survival analysis and several other areas. Second objective is to estimate the unknown model parameters using maximum likelihood method for different sample sizes and different parameter values. To evaluate the performance of the estimators, a simulation study is carried out. In addition, four real life data sets have been analyzed for illustrative purposes. Third objective is to obtain the maximum likelihood estimators (MLEs) of the log Marshall-Olkin Weibull-Weibull (LMOW-W) regression model for censored data to show the flexibility of the log Marshall-Olkin Weibull-Weibull regression model. Thus far we have not come across any report on estimation of parameters for the considered distribution along with regression model for censored data.

We have organized the remainder of this paper in the following way: Section 2 discusses the new MOW-G family. Some sub-models of the family are presented in Section 3 while several statistical properties of this family are given in section 4. Section 5 presents the maximum likelihood estimation for the new family’s parameters. A Monte Carlo simulation study is provided in section 6 to evaluate the performance of the estimators for the MOW-G family. Section 7 illustrates the flexibility of the new family compared to other families using four real data sets. In Section 8, log Marshall-Olkin Weibull-Weibull regression model for censored data is presented. Finally, we conclude the paper in Section 9.

## 2 The MOW-G family

Replacing *G* and *g* in Eqs (1) and (2) by cdf and pdf of the Weibull-G in Eqs (3) and (4), then MOW-G family is obtained. That is, for any baseline distribution with cdf *G*(*x*; ***τ***), the respective MOW-G family associated cdf with parameters ***ζ*** = (*α*, *c*, *β*, ***τ***) is defined as

$$\begin{array}{c} F^{MOWG}\left(x;\zeta \right)=\frac{1-e^{-{\left(\frac{-\text{log}(1-G(x;\tau ))}{\beta }\right)}^{c}}}{1-\bar{\alpha }e^{-{\left(\frac{-\text{log}(1-G(x;\tau ))}{\beta }\right)}^{c}}}. \end{array}$$ (5)

Consequently, its pdf can be obtained as

$$\begin{array}{c} \begin{array}{cc} f^{MOWG}\left(x;\zeta \right) & =\frac{\frac{\alpha c}{\beta }\frac{g(x;\tau )}{1-G(x;\tau )}{\left(\frac{-\text{log}(1-G(x;\tau ))}{\beta }\right)}^{c-1}e^{-{\left(\frac{-\text{log}(1-G(x;\tau ))}{\beta }\right)}^{c}}}{{\left[1-\bar{\alpha }e^{-{\left(\frac{-\text{log}(1-G(x;\tau ))}{\beta }\right)}^{c}}\right]}^{2}}, \end{array} \end{array}$$ (6)

where *α*, *c*, *β* and ***τ*** are defined in Eqs (2) and (4).

## 3 Sub-models of the MOW-G family

Two special distributions are considered as members of the MOW-G family: the MOW-exponential (MOW-E) distribution and the MOW-Weibull (MOW-W) distribution. That is, for the MOW-E distribution, its corresponding cdf and pdf can be correspondingly obtained as

$$\begin{array}{c} F\left(x;\alpha ,c,\beta ,\lambda \right)=\frac{1-e^{-{\left(\frac{\lambda x}{\beta }\right)}^{c}}}{1-\bar{\alpha }e^{-{\left(\frac{\lambda x}{\beta }\right)}^{c}}},\;\text{and}\;f\left(x;\alpha ,c,\beta ,\lambda \right)=\frac{(\frac{\alpha c\lambda }{\beta }){\left(\frac{\lambda x}{\beta }\right)}^{c-1}e^{-{\left(\frac{\lambda x}{\beta }\right)}^{c}}}{{\left[1-\bar{\alpha }e^{-{\left(\frac{\lambda x}{\beta }\right)}^{c}}\right]}^{2}}. \end{array}$$ (7)

where λ > 0 is the scale parameter, *α* and *c*, *β* are defined in (2) and (4), respectively.

Similarly, for the MOW-W distribution, its respective cdf and pdf can be found as

$$\begin{array}{c} F\left(x;\alpha ,c,\beta ,\gamma ,\delta \right)=\frac{1-e^{-{\left({\left(\frac{x}{\delta }\right)}^{\gamma }/\beta \right)}^{c}}}{1-\bar{\alpha }e^{-{\left({\left(\frac{x}{\delta }\right)}^{\gamma }/\beta \right)}^{c}}},\;\text{and}\;f\left(x;\alpha ,c,\beta ,\gamma ,\delta \right)=\frac{\frac{\alpha c\gamma }{\beta^{c}\delta^{\gamma c}}x^{\gamma c-1}e^{-{\left({\left(\frac{x}{\delta }\right)}^{\gamma }/\beta \right)}^{c}}}{{\left[1-\bar{\alpha }e^{-{\left({\left(\frac{x}{\delta }\right)}^{\gamma }/\beta \right)}^{c}}\right]}^{2}}. \end{array}$$ (8)

where *γ* > 0 is the shape parameter, *δ* > 0 is the scale parameter, *α* and *c*, *β* are defined in Eqs (2) and (4), respectively.

**Fig 1:**
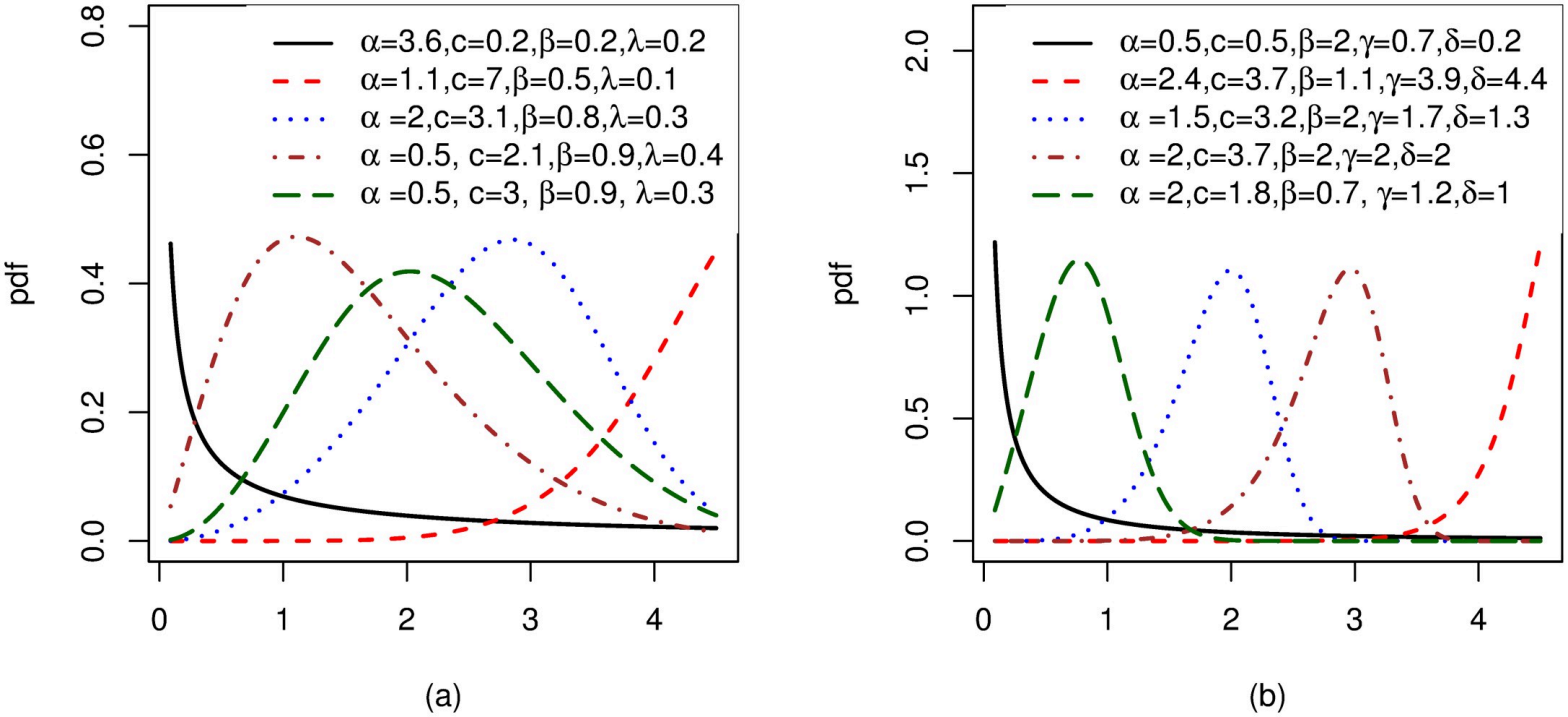
Plots for the pdf of two members from the MOW-G, with different values of the parameters: (a)MOW-E distribution and (b)MOW-W distribution.

**Fig 2:**
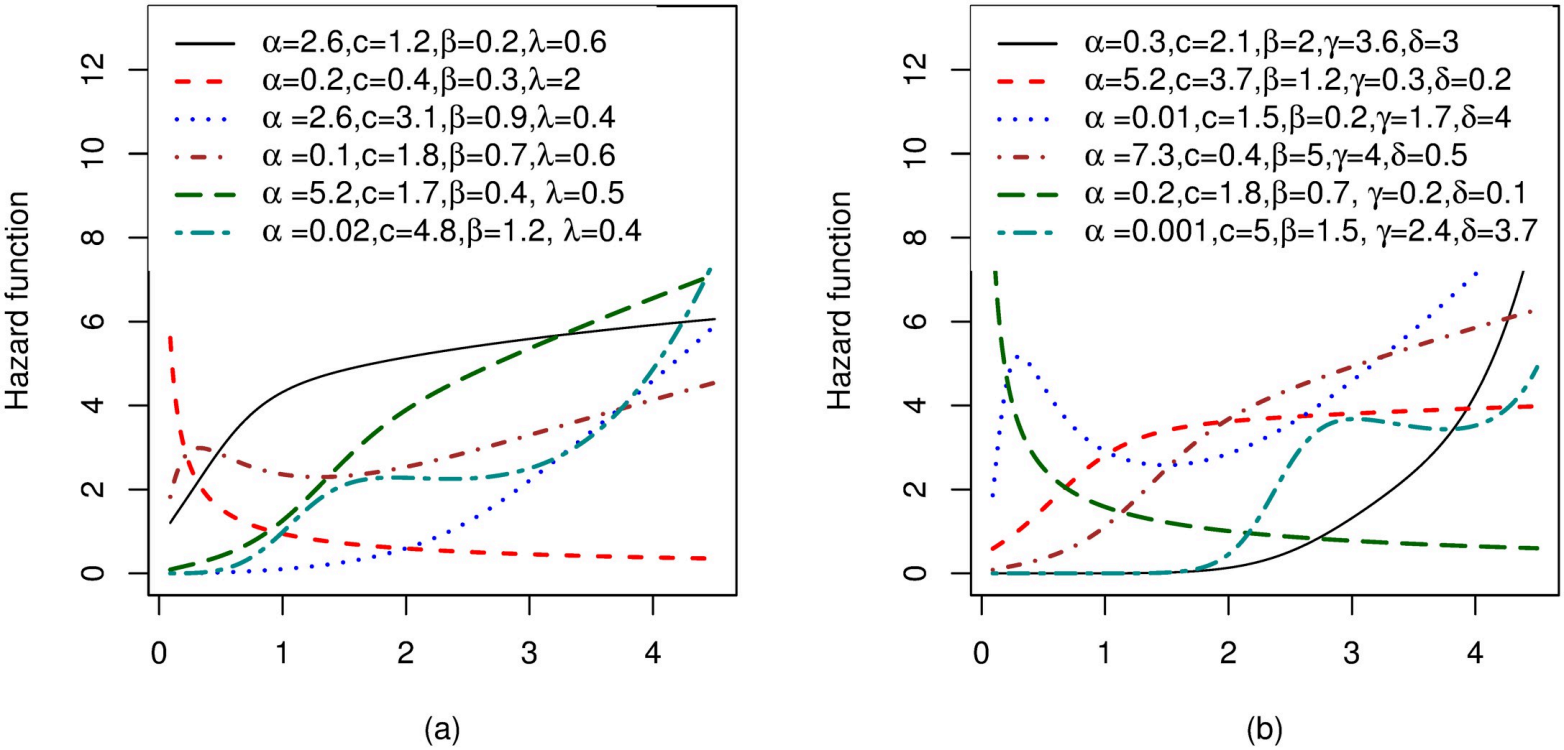
Plots for the hazard function of two members from the MOW-G, with different values of the parameters: (a)MOW-E distribution and (b)MOW-W distribution.

Possible shapes for the density and hazard functions for MOW-E and MOW-W distributions are, respectively shown in Figs 1 and 2. It can be seen from Fig 1 that, the pdf for MOW-E and MOW-W exhibit symmetrical, right-skewed, left-skewed, J shaped and reversed-J shaped densities. Moreover, from Fig 2 we can see that, the hazard for these distributions exhibits increasing, decreasing, bathtub, upside-down bathtub, S shaped, J shaped, and reversed-J shapes. S1 Appendix provides a list of other sub-models associated with the MOW-G family.

## 4 Statistical properties of the MOW-G family

### 4.1 Expansion of the density function

For *α* ∈ (0,1), the binomial expansion states that

$$\begin{array}{c} {\left(1-\omega (x)\right)}^{-k}=\sum_{j=0}^{\infty }\frac{\Gamma (k+j)}{\Gamma (k)j!}\omega {\left(x\right)}^{j}, \end{array}$$ (9)

where Γ(.) is the gamma function.

Then, it follows from Eq (9) that

$$\begin{array}{cc} {\left[1-\bar{\alpha }e^{-{\left(-\frac{\;\text{log}(1-G(x;\tau ))}{\beta }\right)}^{c}}\right]}^{-2} & =\sum_{j=0}^{\infty }\left(j+1\right){\left(1-\alpha \right)}^{j}e^{-j{\left(-\frac{\;\text{log}(1-G(x;\tau ))}{\beta }\right)}^{c}}. \end{array}$$

Therefore, the MOW-G family’s pdf can be obtained as

$$\begin{array}{c} \begin{array}{cc} f^{MOWG}\left(x;\zeta \right)=\sum_{j=0}^{\infty } & \left(j+1\right)\alpha {\left(1-\alpha \right)}^{j}\frac{c}{\beta }\frac{g(x;\tau )}{1-G(x;\tau )}{\left(\frac{-\text{log}(1-G(x;\tau ))}{\beta }\right)}^{c-1}\\ \times & \;e^{-\left(j+1\right){\left(\frac{-\text{log}(1-G(x;\tau ))}{\beta }\right)}^{c}}. \end{array} \end{array}$$

Considering the expansion of the exponential function as

$$\begin{array}{c} e^{-x}=\sum_{i=0}^{\infty }\frac{{\left(-1\right)}^{i}}{i!}x^{i}, \end{array}$$ (10)

we can have,

$$\begin{array}{c} \begin{array}{cc} e^{-\left(j+1\right){\left(\frac{-\text{log}(1-G(x;\tau ))}{\beta }\right)}^{c}} & =\sum_{i=0}^{\infty }\frac{{\left(-1\right)}^{i}}{i!}{\left(j+1\right)}^{i}{\left(\frac{-\text{log}(1-G(x;\tau ))}{\beta }\right)}^{ci}. \end{array} \end{array}$$ (11)

The pdf can be written as

$$\begin{array}{c} \begin{array}{cc} f^{MOWG}\left(x;\zeta \right)=\sum_{j=0}^{\infty }\sum_{i=0}^{\infty } & \frac{{\left(-1\right)}^{i}}{i!}{\left(j+1\right)}^{i+1}\alpha {\left(1-\alpha \right)}^{j}\frac{c}{\beta }\frac{g(x;\tau )}{1-G(x;\tau )}\\ & \times {\left(\frac{-\text{log}(1-G(x;\tau ))}{\beta }\right)}^{c(i+1)-1}. \end{array} \end{array}$$

For *a* > 0, we have the following formula

(−log(1−x))a−1=(a−1)∑m=0∞∑t=0m(−1)t+m(m−a+1m)(mt)Pt,m(a−1−t)xa−1+m, (12)

where the constant *P*_*t*,*m*_ is defined as

$$\begin{array}{c} \begin{array}{cc} P_{t,m} & ={\left(m\right)}^{-1}\sum_{l=1}^{m}\frac{{\left(-1\right)}^{l}[l(t+1)-m]}{l+1}\;P_{t,m-l},\;\text{for}\;m=1,2,\ldots \;\text{and}\;\;P_{t,0}=1\text{.} \end{array} \end{array}$$ (13)

Also, from Eq (9), we have

$$\begin{array}{c} {\left(1-G(x;\tau )\right)}^{-1}=\sum_{r=0}^{\infty }{\left[G(x;\tau )\right]}^{r}. \end{array}$$ (14)

Therefore, from Eqs (12) and (14) and for 0 < *α* < 1, the pdf of MOW-G is given by

fMOWG(x;ζ)=∑j=0∞∑i=0∞∑r=0∞∑m=0∞∑t=0m(−1)i+t+mi!(m−c(i+1)+1m)(mt)Pt,m×c(j+1)i+1α(1−α)j(c(i+1)−1)βc(i+1)(c(i+1)−t−1)(c(i+1)+m+r)×(c(i+1)+m+r)g(x;τ)[G(x;τ)]c(i+1)+m+r−1.

For *α* > 1, the pdf of the MOW-G family can be expressed as follows

$$\begin{array}{c} \begin{array}{cc} f^{MOWG}\left(x;\zeta \right)= & \frac{\alpha c}{\beta }\frac{g(x;\tau )}{1-G(x;\tau )}{\left(\frac{-\text{log}(1-G(x;\tau ))}{\beta }\right)}^{c-1}e^{-{\left(\frac{-\text{log}(1-G(x;\tau ))}{\beta }\right)}^{c}}\\ & \times {\left[\alpha +\bar{\alpha }(1-e^{-{\left(-\frac{\;\text{log}(1-G(x;\tau ))}{\beta }\right)}^{c}})\right]}^{-2}, \end{array} \end{array}$$

which can be written as

$$\begin{array}{c} \begin{array}{cc} f^{MOWG}\left(x;\zeta \right)= & \frac{c}{\beta }\frac{g(x;\tau )}{1-G(x;\tau )}{\left(\frac{-\text{log}(1-G(x;\tau ))}{\beta }\right)}^{c-1}e^{-{\left(\frac{-\text{log}(1-G(x;\tau ))}{\beta }\right)}^{c}}\\ & \times \frac{1}{\alpha }{\left[1-\frac{\left(\alpha -1\right)}{\alpha }(1-e^{-{\left(-\frac{\;\text{log}(1-G(x;\tau ))}{\beta }\right)}^{c}})\right]}^{-2}. \end{array} \end{array}$$

From Eq (9), we can have

$$\begin{array}{c} \begin{array}{cc} {\left[1-\frac{\left(\alpha -1\right)}{\alpha }(1-e^{-{\left(-\frac{\;\text{log}(1-G(x;\tau ))}{\beta }\right)}^{c}})\right]}^{-2} & =\sum_{k=0}^{\infty }(k+1){\left(1-\frac{1}{\alpha }\right)}^{k}{\left(1-e^{-{\left(-\frac{\;\text{log}(1-G(x;\tau ))}{\beta }\right)}^{c}}\right)}^{k}, \end{array} \end{array}$$ (15)

and by using the binomial theorem

(1−x)k=∑j=0k(kj)(−1)jxj, (16)

Eq (15) can be written as

∑k=0∞(k+1)(1−1α)k∑j=0k(kj)(−1)je−j(−log(1−G(x;τ))β)c.

Thus, by replacing $\sum_{k=0}^{\infty }\sum_{j=0}^{k}$ by $\sum_{j=0}^{\infty }\sum_{k=j}^{\infty }$, the MOW-G family’s pdf is expressed as

fMOWG(x;ζ)=∑j=0∞∑k=j∞(−1)j(kj)(k+1)1α(1−1α)kcβg(x;τ)1−G(x;τ)×(−log(1−G(x;τ))β)c−1e−(j+1)(−log(1−G(x;τ))β)c.

Applying Eq (10), the pdf can be given by

fMOWG(x;ζ)=∑j=0∞∑k=j∞∑i=0∞(−1)i+ji!(kj)(k+1)(j+1)i1α(1−1α)kcβc(i+1)×g(x;τ)1−G(x;τ)(−log(1−G(x;τ)))c(i+1)−1.

Now by using Eq (9) for [1 − *G*(*x*; ***τ***)]^−1^ and Eq (12) for (−log(1 − *G*(*x*; ***τ***)))^*c*(*i*+1)−1^, the pdf for MOW-G family with *α* > 1 is obtained by

fMOWG(x;ζ)=∑j=0∞∑k=j∞∑i=0∞∑r=0∞∑m=0∞∑t=0m(−1)i+j+t+mi!(kj)(m−c(i+1)+1m)(mt)Pt,m×c(k+1)(j+1)i1α(1−1α)k(c(i+1)−1)βc(i+1)(c(i+1)−t−1)(c(i+1)+m+r)×(c(i+1)+m+r)g(x;τ)[G(x;τ)]c(i+1)+m+r−1.

Therefore, the pdf of MOW-G family is written as

$$\begin{array}{c} f^{MOWG}\left(x;\zeta \right)=\sum_{j=0}^{\infty }\omega_{j}f^{ExpG}\left(x;c\left(i+1\right)+m+r\right), \end{array}$$ (17)

where

ωj={∑i=0∞∑r=0∞∑m=0∞∑t=0m(−1)i+t+mi!z(j+1)i+1α(1−α)j;0<α<1,∑k=j∞∑i=0∞∑r=0∞∑m=0∞∑t=0m(−1)i+j+t+mi!(kj)z(k+1)(j+1)i1α(1−1α)k;α>1, (18)

and

z=(m−c(i+1)+1m)(mt)Pt,mc(c(i+1)−1)βc(i+1)(c(i+1)−t−1)(c(i+1)+m+r), (19)

for *P*_*t*,*m*_ is defined by (13).

At this point, the cdf and pdf for the exponentiated-G (Exp-G) distribution, denoted by *f*^*ExpG*^(*x*), with a parameter *c* can be respectively defined for an arbitrary *G*(*x*) as

$$\begin{array}{c} F^{ExpG}\left(x\right)={\left[G\left(x\right)\right]}^{c},\;\text{and}\;f^{ExpG}\left(x\right)=c\;g\left(x\right){\left[G\left(x\right)\right]}^{c-1}.\;;\;\text{for}\;c>0 \end{array}$$

Then, a linear combination of the Exp-G density functions serves as a way of expressing the pdf of the MOW-G family. Hence, starting from the properties of the Exp-G class of distributions, some features of the MOW-G family of distributions can be obtained. Such features have been investigated in several studies such as, [34–36], among others.

### 4.2 Moments

Let *Y*_*j*_ is a random variable from Exp-G distribution with parameter (*c*(*i* + 1) + *m* + *r*), then the *r*th moment of the MOW-G family is given by

$$\begin{array}{c} \begin{array}{c} E\left(X^{r}\right)=\int_{0}^{\infty }x^{r}f^{MOWG}\left(x\right)\;dx=\sum_{j=0}^{\infty }\omega_{j}E\left(Y_{j}^{r}\right), \end{array} \end{array}$$

where *ω*_*j*_ is defined in Eq (18).

### 4.3 Moment generating function

The moment generating function of the MOW-G family can be derived in term of the Exp-G distribution as follows

$$\begin{array}{c} \begin{array}{cc} M_{X}\left(t\right) & =E\left(e^{tX}\right)=\int_{0}^{\infty }e^{tx}f^{MOWG}\left(x\right)\;dx=\sum_{j=0}^{\infty }\omega_{j}M_{Y_{j}}\left(t\right), \end{array} \end{array}$$

where *Y*_*j*_ is a random variable from Exp-G distribution with parameter (*c*(*i* + 1) + *m* + *r*) and *ω*_*j*_ is defined in Eq (18).

### 4.4 Incomplete moments

The *s*^*th*^ incomplete moment for MOW-G family is obtained as follows

$$\begin{array}{c} \begin{array}{c} I\left(X^{s}\right)=E\left(X^{s}\right)=\int_{0}^{z}x^{s}f^{MOWG}\left(x\right)\;dx=\sum_{j=0}^{\infty }\omega_{j}I\left(Y_{j}^{s}\right), \end{array} \end{array}$$

where $I(Y_{j}^{s})$ is the incomplete *s*th moment for the Exp-G distribution with parameter (*c*(*i* + 1) + *m* + *r*) and *ω*_*j*_ is defined in Eq (18).

### 4.5 Quantile and median

The quantile function, *Q*(*p*), 0 < *p* < 1, of the MOW-G family is as follows

$$\begin{array}{c} Q\left(p\right)=G^{-1}[1-e^{-\beta {\left(-log(1-\frac{\alpha p}{1-\bar{\alpha }p})\right)}^{\frac{1}{c}}}]. \end{array}$$

Therefore, the median is given by

$$\begin{array}{c} \begin{array}{cc} M= & Q\left(0.5\right)=G^{-1}[1-e^{-\beta {\left(-log(1-\frac{0.5\alpha }{1-0.5\bar{\alpha }})\right)}^{\frac{1}{c}}}]. \end{array} \end{array}$$

### 4.6 Rényi entropy

The Rényi entropy for the MOW-G family is given by

$$\begin{array}{c} H_{R}\left(x\right)=\frac{1}{1-R}\;\text{log}\;[\int_{0}^{\infty }{\left(f^{MOWG}\left(x;\zeta \right)\right)}^{R}\;\;dx]. \end{array}$$

For 0 < *α* < 1, (*f*^*MOWG*^(*x*))^*R*^ can be obtained using Eq (9) as

$$\begin{array}{c} \begin{array}{cc} {\left[f^{MOWG}\left(x;\zeta \right)\right]}^{R}= & {\left(\frac{\alpha c}{\beta }\right)}^{R}{\left(\frac{g(x;\tau )}{1-G(x;\tau )}\right)}^{R}{\left(\frac{-\text{log}(1-G(x;\tau ))}{\beta }\right)}^{R(c-1)}\\ \times & \;e^{-R{\left(\frac{-\text{log}(1-G(x;\tau ))}{\beta }\right)}^{c}}\;\sum_{j=0}^{\infty }\frac{\Gamma (2R+j)}{\Gamma (2R)j!}{\bar{\alpha }}^{j}e^{-j{\left(\frac{-\text{log}(1-G(x;\tau ))}{\beta }\right)}^{c}}. \end{array} \end{array}$$

Therefore,

$$\begin{array}{c} \begin{array}{c} H_{R}\left(x\right)=\frac{1}{1-R}[R\;\text{log}(\frac{\alpha c}{\beta })+\;\text{log}(\sum_{j=0}^{\infty }\frac{\Gamma (2R+j)}{\Gamma (2R)j!}{\left(1-\alpha \right)}^{j}A)], \end{array} \end{array}$$

where

$$\begin{array}{c} \begin{array}{cc} A & =\int_{0}^{\infty }{\left(\frac{g(x;\tau )}{1-G(x;\tau )}\right)}^{R}{\left(\frac{-\text{log}(1-G(x;\tau ))}{\beta }\right)}^{R(c-1)}e^{-\left(j+R\right){\left(\frac{-\text{log}(1-G(x;\tau ))}{\beta }\right)}^{c}}dx. \end{array} \end{array}$$ (20)

Now, using Eq (10), we have

$$\begin{array}{c} \begin{array}{cc} A & =\frac{1}{\beta^{R(c-1)+ci}}\sum_{i=0}^{\infty }\frac{{\left(-1\right)}^{i}}{i!}{\left(j+R\right)}^{i}\int_{0}^{\infty }{\left(\frac{g(x;\tau )}{1-G(x;\tau )}\right)}^{R}{\left(-\text{log}(1-G(x;\tau ))\right)}^{R(c-1)+ci}\;dx. \end{array} \end{array}$$

Applying Eqs (9) and (12), respectively, for (1 − *G*(*x*; ***τ***))^−*R*^ and (−log(1 − *G*(*x*; ***τ***)))^*R*(*c*−1)+*ci*^, the Rényi entropy for the MOW-G family for *α* ∈ (0, 1), is expressed as

A=1βR(c−1)+ci∑i=0∞∑r=0∞∑m=0∞∑t=0m(−1)i+t+mi!(j+R)iΓ(R+r)Γ(R)r!(R(c−1)+ci)×(m−R(c−1)−cim)(mt)Pt,mR(c−1)+ci−t∫0∞(g(x;τ)R[G(x;τ)]R(c−1)+ci+m+rdx. (21)

HR(x)=R1−Rlog(αcβ)+11−Rlog(∑j=0∞∑i=0∞∑r=0∞∑m=0∞∑t=0m(−1)i+t+mi!(m−R(c−1)−cim)(mt)Pt,m×(1−α)j(j+R)iΓ(2R+j)Γ(R+r)(R(c−1)+ci)βR(c−1)+ciΓ(2R)Γ(R)r!j!(R(c−1)+ci−t)×RR(c(i+R)+m+r)Re(1−R)HRExpG), (22)

where Γ(.) is the gamma function, *P*_*t*,*m*_ is defined in Eq (13) and $H_{R}^{ExpG}$ is the Rényi entropy for the Exp-G distribution with parameter ($\frac{c(i+R)+m+r}{R}$).

Now for *α* > 1 and using Eq (9), we have

$$\begin{array}{c} \begin{array}{cc} {\left[f^{MOWG}\left(x;\zeta \right)\right]}^{R} & ={\left(\frac{c}{\alpha \beta }\right)}^{R}{\left(\frac{g(x;\tau )}{1-G(x;\tau )}\right)}^{R}{\left(\frac{-\text{log}(1-G(x;\tau ))}{\beta }\right)}^{R(c-1)}e^{-R{\left(\frac{-\text{log}(1-G(x;\tau ))}{\beta }\right)}^{c}}\\ & \times \sum_{k=0}^{\infty }\frac{\Gamma (2R+k)}{\Gamma (2R)k!}{\left(\frac{\alpha -1}{\alpha }\right)}^{k}{\left(1-e^{-{\left(\frac{-\text{log}(1-G(x;\tau ))}{\beta }\right)}^{c}}\right)}^{k}. \end{array} \end{array}$$

Applying Eq (16), and replacing $\sum_{k=0}^{\infty }\sum_{j=0}^{k}$ by $\sum_{j=0}^{\infty }\sum_{k=j}^{\infty }$, the entropy is written as

HR(x)=11−R[Rlog(cαβ)+log(∑j=0∞∑k=j∞(−1)j(kj)Γ(2R+k)Γ(2R)k!(1−1α)kA)],

where *A* is defined in Eq (20).

Thus, for *α* > 1, the Rényi entropy for the MOW-G family is written as

HR(x)=R1−Rlog(cαβ)+11−Rlog(∑j=0∞∑k=j∞∑i=0∞∑r=0∞∑m=0∞∑t=0m(−1)i+j+t+mi!(kj)(m−R(c−1)−cim)(mt)×Pt,m(1−1α)k(j+R)iΓ(2R+k)Γ(R+r)(R(c−1)+ci)βR(c−1)+ciΓ(2R)Γ(R)k!r!(R(c−1)+ci−t)×RR(c(i+R)+m+r)Re(1−R)HRExpG),

where Γ(.) is the gamma function, *P*_*t*,*m*_ is defined in Eq (13) and $H_{R}^{ExpG}$ is the the Rényi entropy for the Exp-G distribution with parameter ($\frac{c(i+R)+m+r}{R}$).

### 4.7 Distribution of order statistics

Consider the random sample *x*_1_, *x*_2_, …, *x*_*n*_ from MOW-G distribution with order statistics *x*_1:*n*_ < *x*_2:*n*_ < … < *x*_*n*:*n*_. Then the distribution of the *q*th order statistics of MOW-G distribution is given by

$$\begin{array}{c} \begin{array}{cc} f_{q:n}^{MOWG}\left(x;\zeta \right) & =\frac{n!}{(q-1)!(n-q)!}f^{MOWG}\left(x;\zeta \right){\left[F^{MOWG}\left(x;\zeta \right)\right]}^{q-1}{\left[1-F^{MOWG}\left(x;\zeta \right)\right]}^{n-q}, \end{array} \end{array}$$

where from Eq (16),

[1−FMOWG(x;ζ)]n−q=∑p=0n−q(n−qp)(−1)p[FMOWG(x;ζ)]p.

Then, for for *α* ∈ (0, 1) and after some algebra, the distribution of the *q*th order statistics of the MOW-G is expressed as follows

fq:nMOWG(x;ζ)=∑p=0n−q∑s=0p+q−1∑j=0∞∑i=0∞∑r=0∞∑m=0∞∑t=0m(−1)p+s+i+t+mi!n!(q−1)!(n−q)!×z(n−qp)(p+q−1s)α(1−α)j(j+s+1)iΓ(p+q+j+1)Γ(p+q+1)j!×(c(i+1)+m+r)g(x;τ)[G(x;τ)]c(i+1)+m+r−1. (24)

Similarly, for *α* > 1, we can obtain

fq:nMOWG(x;ζ)=∑p=0n−q∑k=0∞∑j=0p+q+k−1∑i=0∞∑r=0∞∑m=0∞∑t=0m(−1)p+j+i+t+mi!n!(q−1)!(n−q)!×z(n−qp)(p+q+k−1j)(1α)p+q(1−1α)k(j+1)iΓ(p+q+k+1)Γ(p+q+1)k!×(c(i+1)+m+r)g(x;τ)[G(x;τ)]c(i+1)+m+r−1, (25)

where Γ(.) is the gamma function and *z* is defined in Eq (19).

From Eqs (24) and (25), we can see that the pdf for the order statistic of the MOW-G family is an infinite linear combination of the pdf of the Exp-G with parameter (*c*(*i* + 1) + *m* + *r*). Thus, many proprieties of these order statistics can be achieved from the proprieties of the Exp-G.

## 5 MOW-G family’s parameters estimation

To obtain the estimators for the parameters of any member from the MOW-G family, the maximum likelihood method can be applied. That is, if we have *x*_1_, *x*_2_, …, *x*_*n*_ follows a distribution associated to the MOW-G class, with a parameters vector ***φ*** = (*α*, *β*, *c*, ***τ***), where ***τ*** represents the vector of parameters of the baseline distribution G, then the log-likelihood function (*ℓ*) is defined by

$$\begin{array}{c} \begin{array}{cc} \ell & =n\;\text{log}\;\alpha +n\;\text{log}\;c-nc\;\text{log}\;\beta +\sum_{i=1}^{n}\;\text{log}(g(x_{i};\tau ))-\sum_{i=1}^{n}\;\text{log}(1-G(x_{i};\tau ))\\ & +\left(c-1\right)\sum_{i=1}^{n}\;\text{log}(-\text{log}(1-G\left(x_{i};\tau \right)))-\sum_{i=1}^{n}{\left(\frac{-\text{log}(1-G\left(x_{i};\tau \right))}{\beta }\right)}^{c}\\ & -2\sum_{i=1}^{n}\;\text{log}\;[1-\bar{\alpha }e^{-{\left(\frac{-\text{log}(1-G\left(x_{i};\tau \right))}{\beta }\right)}^{c}}]. \end{array} \end{array}$$ (26)

The complexity of the above log-likelihood makes it difficult to solve analytically. Therefore, we take the derivative of Eq (26) with respect to the parameters as follows

$$\begin{array}{c} \frac{\partial \ell }{\partial \alpha }=\frac{n}{\alpha }-2\sum_{i=1}^{n}\frac{e^{-{\left(\frac{-\text{log}(1-G\left(x_{i};\tau \right))}{\beta }\right)}^{c}}}{\left[1-\bar{\alpha }e^{-{\left(\frac{-\text{log}(1-G\left(x_{i};\tau \right))}{\beta }\right)}^{c}}\right]}, \end{array}$$

$$\begin{array}{c} \begin{array}{cc} & \frac{\partial \ell }{\partial c}=\frac{n}{c}-n\;\text{log}\;\beta +\sum_{i=1}^{n}\;\text{log}(-\text{log}(1-G\left(x_{i};\tau \right)))\\ & -\sum_{i=1}^{n}\;\text{log}(\frac{-\text{log}(1-G\left(x_{i};\tau \right))}{\beta }){\left(\frac{-\text{log}(1-G\left(x_{i};\tau \right))}{\beta }\right)}^{c}\\ & +2\bar{\alpha }\sum_{i=1}^{n}\frac{\;\text{log}(\frac{-\text{log}(1-G\left(x_{i};\tau \right)}{\beta }){\left(\frac{-\text{log}(1-G\left(x_{i};\tau \right))}{\beta }\right)}^{c}}{\bar{\alpha }-e^{{\left(\frac{-\text{log}(1-G(x,\tau ))}{\beta }\right)}^{c}}}, \end{array} \end{array}$$

$$\begin{array}{c} \begin{array}{cc} \frac{\partial \ell }{\partial \beta } & =-\frac{nc}{\beta }+\frac{c}{\beta }\sum_{i=1}^{n}{\left(\frac{-\text{log}(1-G\left(x_{i};\tau \right))}{\beta }\right)}^{c}+2\bar{\alpha }c\sum_{i=1}^{n}\frac{{\left(\frac{-log(1-G\left(x_{i};\tau \right))}{\beta }\right)}^{c}}{\beta (e^{{\left(\frac{-\text{log}(1-G\left(x_{i};\tau \right))}{\beta }\right)}^{c}}-\bar{\alpha })}, \end{array} \end{array}$$

and

$$\begin{array}{c} \begin{array}{cc} \frac{\partial \ell }{\partial \tau } & =n\;\text{log}\;\alpha +n\;\text{log}\;c-nc\;\text{log}\;\beta +\frac{\partial }{\partial \tau }\sum_{i=1}^{n}\;\text{log}\;\left(g\left(x_{i};\tau \right)\right)-\frac{\partial }{\partial \tau }\sum_{i=1}^{n}\;\text{log}\;\left(1-G\left(x_{i};\tau \right)\right)\\ & +\frac{\partial }{\partial \tau }\left(c-1\right)\sum_{i=1}^{n}\;\text{log}(\frac{-\text{log}(1-G\left(x_{i};\tau \right))}{\beta })-\frac{\partial }{\partial \tau }\sum_{i=1}^{n}{\left(\frac{-\text{log}(1-G\left(x_{i};\tau \right))}{\beta }\right)}^{c}\\ & -2\frac{\partial }{\partial \tau }\sum_{i=1}^{n}\;\text{log}\;[1-\bar{\alpha }e^{-{\left(\frac{-log(1-G\left(x_{i};\tau \right))}{\beta }\right)}^{c}}]. \end{array} \end{array}$$

Thus, the MLEs of the parameters vector ***φ*** can be acquired by obtaining the solutions in iterative way of the above nonlinear equations using the well-known Newton-Raphson method or any other numerical method. Instead, employing any standard non-linear optimization technique, the log-likelihood in Eq (26) can be directly maximized.

## 6 Simulation study

For evaluating the performance of the MLEs, some simulation studies is conducted for the two particular members of the MOW-G family namely; MOW-E distribution with rate parameter λ and MOW-W distribution with shape parameter *γ* and scale parameter *δ*. For each distribution, the simulation is performed over the number of iterations, *nsimu* = 1000. Four distinct sample sizes *n* = 25, 50, 100, 200, 500 are considered with the following cases for the true parameters, *θ*_*tru*_.

- **For MOW-E**:
  $$\begin{array}{c} \begin{array}{cc} & \text{Case}\;\text{I}:\;\alpha =0.05,\;c=3,\;\beta =0.2,\;\lambda =0.7\\ & \text{Case}\;\text{II}:\;\alpha =0.05,\;c=3,\;\beta =0.2,\;\lambda =0.7\\ & \text{Case}\;\text{III}:\;\alpha =1.3,\;c=1.3,\;\beta =2.2,\;\lambda =1.2\\ & \text{Case}\;\text{IV}:\;\alpha =2.2,\;c=1.2,\;\beta =1.3,\lambda =2.0 \end{array} \end{array}$$
- **For MOW-W**:
  $$\begin{array}{c} \begin{array}{cc} & \text{Case}\;\text{I}:\;\alpha =0.03,\;c=1.2,\;\beta =0.3,\gamma =0.7,\delta =0.5\\ & \text{Case}\;\text{II}:\;\alpha =0.02,\;c=1.6,\;\beta =0.4,\gamma =1.2,\delta =0.5\\ & \text{Case}\;\text{III}:\;\alpha =1.2,\;c=2.6,\;\beta =1.4,\gamma =2.0,\delta =1.5\\ & \text{Case}\;\text{IV}:\;\alpha =2.2,\;c=5.2,\;\beta =2.2,\gamma =0.9,\delta =2.0 \end{array} \end{array}$$

We compute the mean square errors (MSEs) and biases of the MLEs of the parameters based on 1000 iterations, where

$$\begin{array}{c} bias\left(\hat{\theta }\right)=\frac{\sum_{i=1}^{nsimu}{\hat{\theta }}_{i}}{nsimu}-\theta_{tru},\;\text{and}\;RMSE\left(\hat{\theta }\right)=\sqrt{\frac{\sum_{i=1}^{nsimu}{\left({\hat{\theta }}_{i}-\theta_{tru}\right)}^{2}}{nsimu}}. \end{array}$$

The R programming language [37] has been used to conduct the Monte Carlo simulation studies. Tables 1 and 2 report the results for the MLEs for parameters of the MOW-E and MOW-W along with their corresponding absolute average bias and RMSE, respectively.

**Table 1:** MOW-E parameter estimates, absolute bias and RMSE for four different cases.

		**Case I**			**Case II**		
		MLE	Bias	RMSE	MLE	Bias	RMSE
*n* = 25	*α*	0.6605	0.6105	4.3207	0.8953	0.8853	5.6300
	*c*	2.9519	0.0481	0.6663	5.1535	0.1465	1.1218
	*β*	0.3055	0.1055	0.1745	4.6348	2.2348	3.3710
	λ	1.7182	1.0182	1.8956	2.2666	1.5666	2.5822
*n* = 50	*α*	0.4057	0.3557	3.6130	0.1804	0.1704	1.6460
	*c*	2.9579	0.0421	0.4778	5.1761	0.1239	0.7576
	*β*	0.2623	0.0623	0.1370	4.2898	1.8898	3.1044
	λ	1.2951	0.5951	1.3514	1.8270	1.1270	1.9590
*n* = 100	*α*	0.1731	0.1231	2.3818	0.0447	0.0347	0.0700
	*c*	2.9791	0.0209	0.3468	5.2161	0.0839	0.4930
	*β*	0.2530	0.0530	0.1069	3.6792	1.2792	2.2235
	λ	1.0719	0.3719	0.8695	1.3796	0.6796	1.1945
*n* = 200	*α*	0.0757	0.0257	0.0630	0.0275	0.0175	0.0330
	*c*	2.9775	0.0225	0.2286	5.2268	0.0732	0.3411
	*β*	0.2338	0.0338	0.0852	3.3813	0.9813	1.6353
	λ	0.9129	0.2129	0.4999	1.1685	0.4685	0.7964
*n* = 500	*α*	0.0599	0.0099	0.0313	0.0173	0.0073	0.0138
	*c*	2.9901	0.0099	0.1417	5.2531	0.0469	0.2059
	*β*	0.2241	0.0241	0.0516	2.8751	0.4751	0.8983
	λ	0.8206	0.1206	0.2779	0.9186	0.2186	0.4102
		**Case III**			**Case IV**		
		MLE	Bias	RMSE	MLE	Bias	RMSE
*n* = 25	*α*	0.9127	0.3873	0.8674	1.2584	0.9416	1.5140
	*c*	1.4706	0.1706	0.5572	1.4466	0.2466	0.5936
	*β*	2.1346	0.0654	0.4746	1.3103	0.0103	0.4435
	λ	1.1740	0.0260	1.0423	2.0337	0.0337	1.1219
*n* = 50	*α*	1.0095	0.2905	0.8551	1.5321	0.6679	1.4188
	*c*	1.3787	0.0787	0.4468	1.3220	0.1220	0.4439
	*β*	2.0948	0.1052	0.3952	1.3043	0.0043	0.3937
	λ	1.1530	0.0470	0.9594	2.1583	0.1583	1.1172
*n* = 100	*α*	1.0936	0.2064	0.7166	1.7134	0.4866	1.2383
	*c*	1.3245	0.0245	0.3276	1.2422	0.0422	0.3508
	*β*	2.1689	0.0311	0.3917	1.2551	0.0449	0.3751
	λ	1.3357	0.1357	0.8797	2.2024	0.2024	1.0752
*n* = 200	*α*	1.2229	0.0771	0.6552	1.8926	0.3074	1.1067
	*c*	1.3174	0.0174	0.2367	1.2101	0.0101	0.2710
	*β*	2.1157	0.0843	0.2913	1.2568	0.0432	0.2679
	λ	1.2856	0.0856	0.5987	2.184	0.184	0.893
*n* = 500	*α*	1.3458	0.0458	0.5634	2.2098	0.0098	0.9629
	*c*	1.3081	0.0081	0.1478	1.2057	0.0057	0.1798
	*β*	2.2513	0.0513	0.1988	1.2655	0.0345	0.2110
	λ	1.2988	0.0988	0.3981	2.0706	0.0706	0.6720

**Table 2:** MOW-W parameter estimates, absolute bias and RMSE for four different cases.

		**Case I**			**Case II**		
		MLE	Bias	RMSE	MLE	Bias	RMSE
*n* = 25	*α*	0.4298	0.3998	1.5722	0.4929	0.4729	2.1199
	*c*	1.2314	0.0314	0.1771	1.5819	0.0181	0.2118
	*β*	0.2297	0.0703	0.1420	0.3052	0.0948	0.1472
	*γ*	0.6594	0.0406	0.1056	1.1690	0.0310	0.1489
	*δ*	0.3758	0.1242	0.2553	0.3643	0.1357	0.1938
*n* = 50	*α*	0.2424	0.2124	1.3979	0.2463	0.2263	1.7105
	*c*	1.2138	0.0138	0.1271	1.5822	0.0178	0.1596
	*β*	0.2518	0.0482	0.1181	0.3348	0.0652	0.1191
	*γ*	0.6747	0.0253	0.0848	1.1757	0.0243	0.1128
	*δ*	0.4116	0.0884	0.2176	0.4112	0.0888	0.1600
*n* = 100	*α*	0.0896	0.0596	0.5513	0.0596	0.0396	0.0869
	*c*	1.2122	0.0122	0.0882	1.5798	0.0202	0.1136
	*β*	0.2740	0.0260	0.0999	0.3583	0.0417	0.0903
	*γ*	0.6885	0.0115	0.0556	1.1950	0.0050	0.0672
	*δ*	0.4529	0.0471	0.1754	0.4437	0.0563	0.1175
*n* = 200	*α*	0.0488	0.0188	0.0451	0.0382	0.0182	0.0388
	*c*	1.2075	0.0075	0.0526	1.5977	0.0023	0.0829
	*β*	0.2852	0.0148	0.0766	0.3746	0.0254	0.0683
	*γ*	0.6926	0.0074	0.0332	1.1892	0.0108	0.0560
	*δ*	0.4762	0.0238	0.1313	0.4665	0.0335	0.0940
*n* = 500	*α*	0.0380	0.0080	0.0214	0.0275	0.0075	0.0173
	*c*	1.2003	0.0003	0.0443	1.5971	0.0029	0.0601
	*β*	0.2912	0.0088	0.0557	0.3862	0.0138	0.0502
	*γ*	0.6984	0.0016	0.0289	1.1978	0.0022	0.0366
	*δ*	0.4868	0.0132	0.0955	0.4802	0.0198	0.0625
		**Case III**			**Case IV**		
		MLE	Bias	RMSE	MLE	Bias	RMSE
*n* = 25	*α*	0.8979	0.3021	1.1745	1.3912	0.8088	1.5044
	*c*	2.6101	0.0101	0.5369	5.5555	0.3555	1.0127
	*β*	1.3456	0.0544	0.2219	2.2709	0.0709	0.3439
	*γ*	2.1880	0.1880	0.5499	1.0184	0.1184	0.3785
	*δ*	1.5420	0.0420	0.2822	2.1998	0.1998	0.7712
*n* = 50	*α*	0.9547	0.2453	1.1362	1.5113	0.6887	1.3924
	*c*	2.4866	0.1134	0.4820	5.4433	0.2433	0.9585
	*β*	1.3067	0.0933	0.2180	2.2159	0.0159	0.2595
	*γ*	2.1758	0.1758	0.4939	0.9641	0.0641	0.3227
	*δ*	1.5485	0.0485	0.2666	2.1069	0.1069	0.7169
*n* = 100	*α*	1.0478	0.1522	0.9830	1.7358	0.4642	1.2364
	*c*	2.5888	0.0112	0.4099	5.2056	0.0056	0.8799
	*β*	1.3759	0.0241	0.1797	2.1512	0.0488	0.2555
	*γ*	2.0496	0.0496	0.3711	0.9732	0.0732	0.2546
	*δ*	1.4991	0.0009	0.2101	2.1877	0.1877	0.6412
*n* = 200	*α*	1.3463	0.1463	0.8991	1.9533	0.2467	1.0816
	*c*	2.5743	0.0257	0.3129	5.4280	0.2280	0.7116
	*β*	1.3721	0.0279	0.1293	2.1890	0.0110	0.1599
	*γ*	2.0329	0.0329	0.2963	0.8984	0.0016	0.1943
	*δ*	1.5021	0.0021	0.1626	1.9947	0.0053	0.4597
*n* = 500	*α*	1.2030	0.0030	0.4509	2.2156	0.0156	0.9724
	*c*	2.6267	0.0267	0.1738	5.1682	0.0318	0.5765
	*β*	1.4056	0.0056	0.0827	2.1814	0.0186	0.1417
	*γ*	1.9997	0.0003	0.1984	0.9208	0.0208	0.1525
	*δ*	1.4963	0.0037	0.1108	2.0508	0.0508	0.3866

The “optim” function in R software is used to achieve all estimation results. The results of the simulation study are reported in Tables 1 and 2. Tables 1 and 2 show that the RMSEs decrease as the sample size increases for all parameters. The bias tend to zero for large n, that is, the estimates are asymptotically unbiased. The RMSEs tend to zero as *n* increases, which indicates that the estimates are consistent. This provides evidence that the maximum likelihood method has a good performance when estimating the parameters of the proposed family.

## 7 Applications

Four real-world data sets with the exponential and Weibull distributions as the G, are considered to investigate the flexibility of the proposed MOW-G family. The first data set is fitted to MOW-E distribution and compared with Marshall Olkin-G defined by [2, 38] and Weibull-G defined by [39] considering G as exponential and Weibull. Second and third data sets are also fitted to MO extended Weibull (MOEW) defined by [6]. While the fourth data set is fitted to MOW-W distribution and compared with MO-exponential (MO-E), Weibull-G and Exp-G defined by [1] considering G as exponential and Weibull.

The proposed model is compared with other models according to some criteria, include the value of the log likelihood function (*ℓ*), Akaike information criterion (AIC), Bayesian information criterion (BIC), consistent Akaike information criterion (CAIC), Cramér-von Mises (W*), Anderson-Darling (A*), Kolmogorov-Smirnov (KS) and P-value statistics. These statistics or criteria are widely used to assess the performance of a distribution in modeling a data set. Smaller values of these statistics, indicate a better fit.

**Data set I**: The data represents COVID-19 drought mortality rate of Canada for 36 days, in the period 10 April to 15 May 2020, at https://covid19.who.int. The data is obtained from [40] and listed in Table 3.

**Table 3:** List of data set I.

3.1091	3.3825	3.1444	3.2135	2.4946	3.5146	4.9274	3.3769	6.8686
3.0914	4.9378	3.1091	3.2823	3.8594	4.0480	4.1685	3.6426	3.2110
2.8636	3.2218	2.9078	3.6346	2.7957	4.2781	4.2202	1.5157	2.6029
3.3592	2.8349	3.1348	2.5261	1.5806	2.7704	2.1901	2.4141	1.9048

**Data set II**: Represents tde number of successive failures for tde air conditioning system of each member in a fleet of 13 Boeing 720 jet airplanes [41]. Tde data is listed in Table 4.

**Table 4:** List of data set II.

194	413	90	74	55	23	97	50	359	50	130	487
57	102	15	14	10	57	320	261	51	44	9	254
493	33	18	209	41	58	60	48	56	87	11	102
12	5	14	14	29	37	186	29	104	7	4	72
270	283	7	61	100	61	502	220	120	141	22	603
35	98	54	100	11	181	65	49	12	239	14	18
39	3	12	5	32	9	438	43	134	184	20	386
182	71	80	188	230	152	5	36	79	59	33	246
1	79	3	27	201	84	27	156	21	16	88	130
14	118	44	15	42	106	46	230	26	59	153	104
20	206	5	66	34	29	26	35	5	82	31	118
326	12	54	36	34	18	25	120	31	22	18	216
139	67	310	3	46	210	57	76	14	111	97	62
39	30	7	44	11	63	23	22	23	14	18	13
34	16	18	130	90	163	208	1	24	70	16	101
52	208	95	62	11	191	14	71				

**Data set III**: Represents strengtd data measured in GPA for single carbon fibers which were tested under tension at gauge lengtds of 20 mm and impregnated 1000-carbon fiber tows. [42] originally reported tdis data witd 63 observations in Table 5.

**Table 5:** List of data set III.

1.901	2.132	2.203	2.228	2.257	2.350	2.361	2.396
2.397	2.445	2.454	2.474	2.518	2.522	2.525	2.532
2.575	2.614	2.616	2.618	2.624	2.659	2.675	2.738
2.740	2.856	2.917	2.928	2.937	2.937	2.977	2.996
3.030	3.125	3.139	3.145	3.220	3.223	3.235	3.243
3.264	3.272	3.294	3.332	3.346	3.377	3.408	3.435
3.493	3.501	3.537	3.554	3.562	3.628	3.852	3.871
3.886	3.971	4.024	4.027	4.225	4.395	5.020	

**Data set IV**: Represents experimental data of tde strengtd of glass fibers witd lengtds 1.5 cm. Tde source of such data is tde National Physical Laboratory in England and it is composed of 63 observations and listed in Table 6, [43].

**Table 6:** List of data set IV.

0.55	0.93	1.25	1.36	1.49	1.52	1.58	1.61	1.64	1.68	1.73
1.81	2	0.74	1.04	1.27	1.39	1.49	1.53	1.59	1.61	1.66
1.68	1.76	1.82	2.01	0.77	1.11	1.28	1.42	1.5	1.54	1.6
1.62	1.66	1.69	1.76	1.84	2.24	0.81	1.13	1.29	1.48	1.5
1.55	1.61	1.62	1.66	1.7	1.77	1.84	0.84	1.24	1.3	1.48
1.51	1.55	1.61	1.63	1.67	1.7	1.78	1.89			

**Fig 3:**
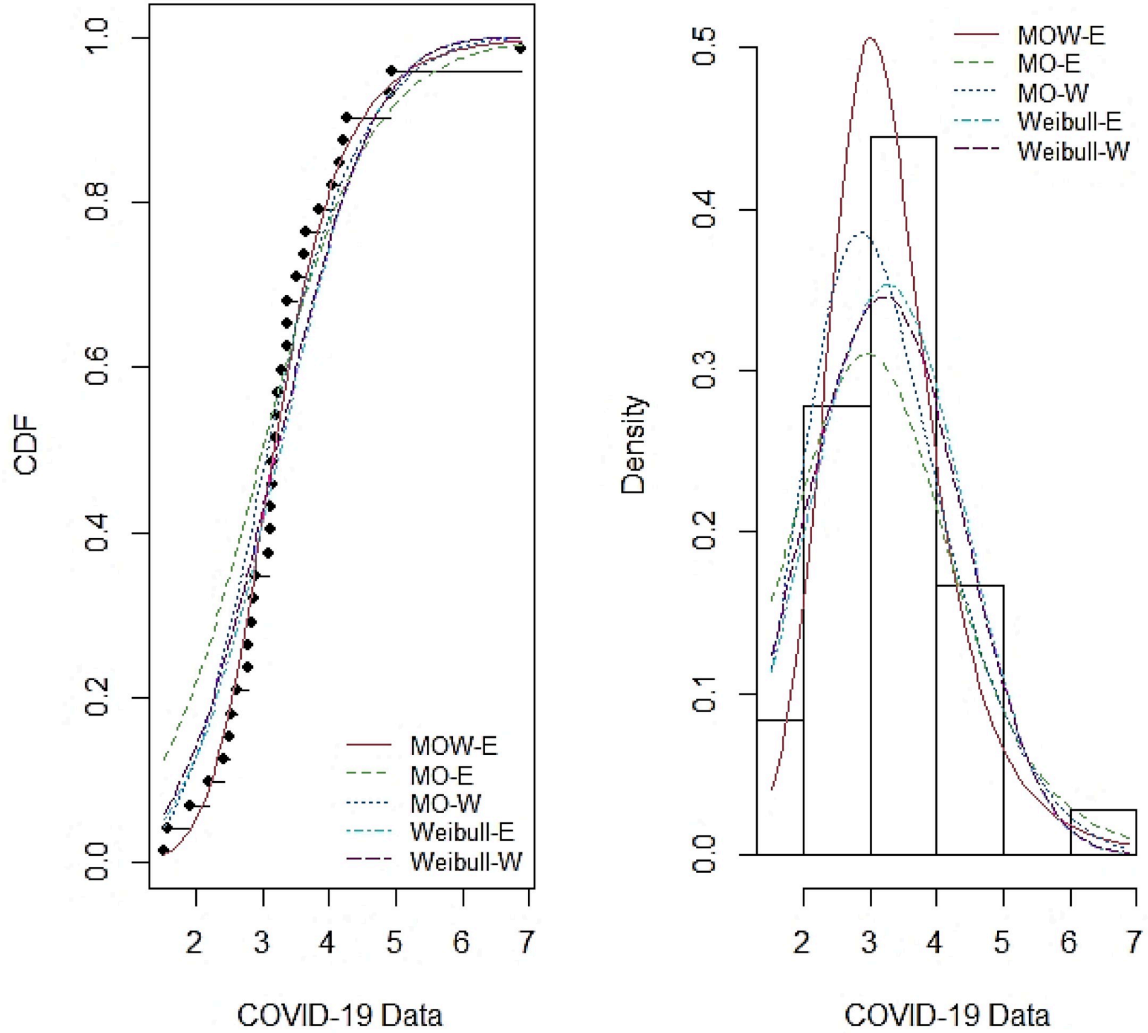
Estimated pdf and cdf of MOW-E and other competing distributions for data set I.

**Fig 4:**
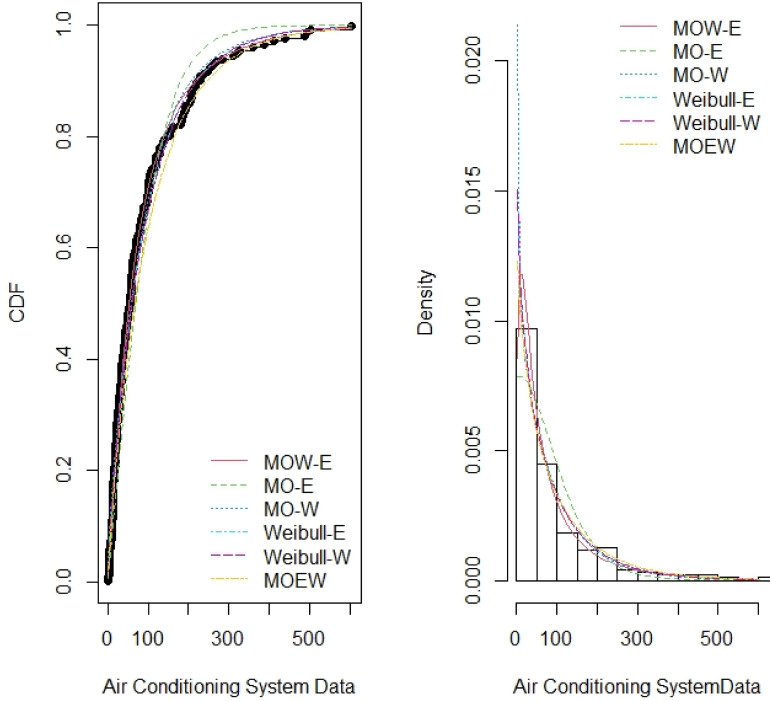
Estimated pdf and cdf of MOW-E and other competing distributions for data set II.

**Fig 5:**
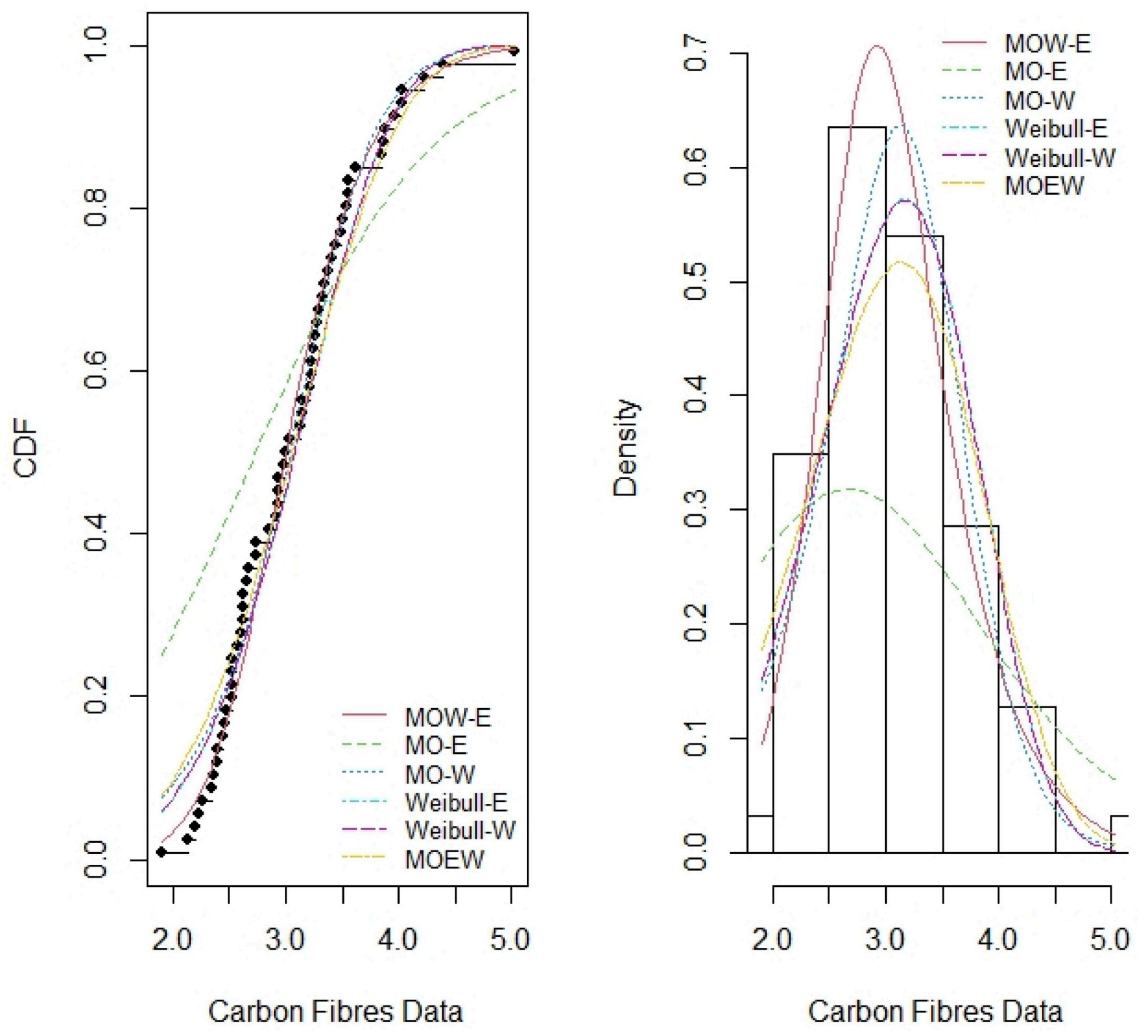
Estimated pdf and cdf of MOW-E and other competing distributions for data set III.

**Fig 6:**
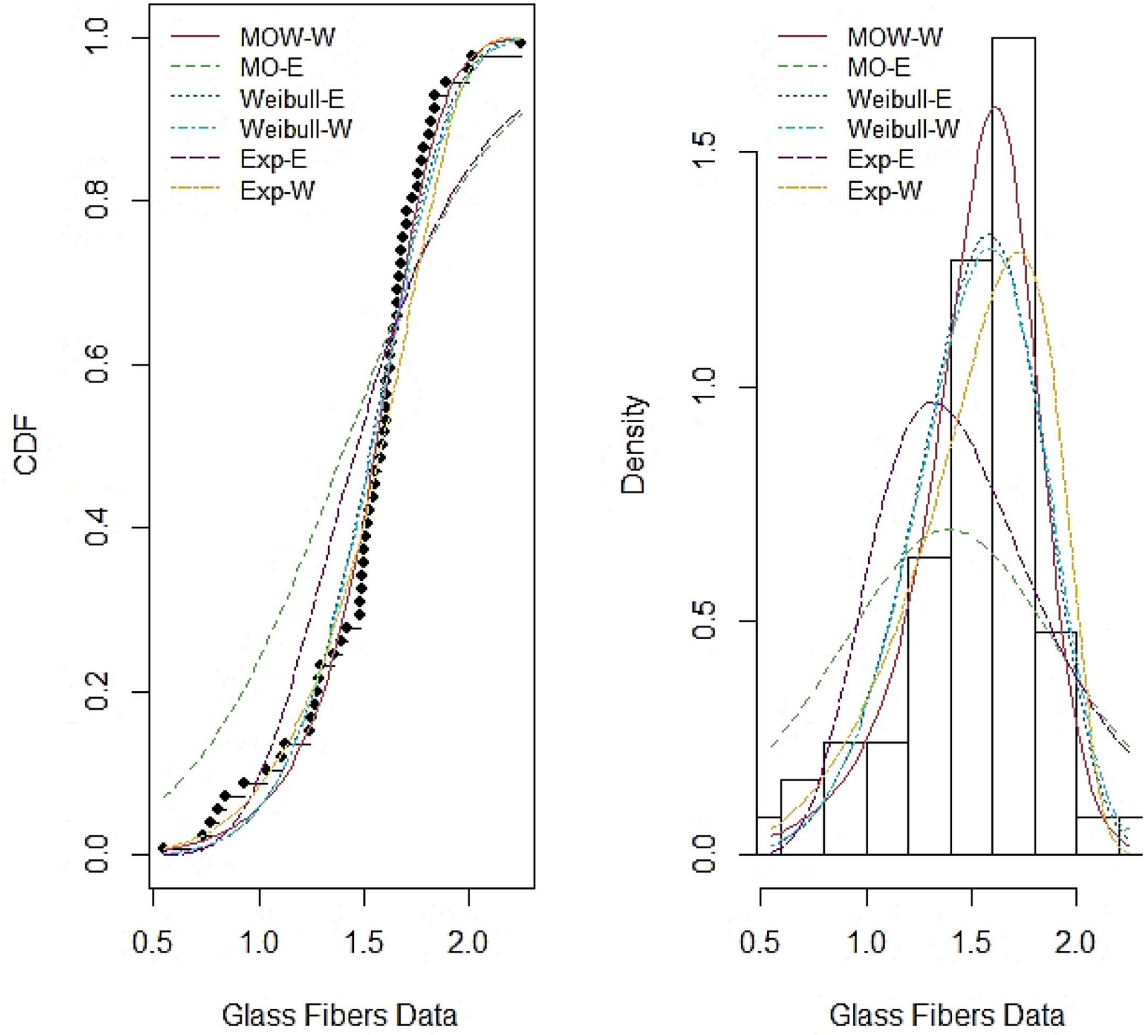
Estimated pdf and cdf of MOW-W and other competing distributions for data set IV.

Tables 7–14 report the MLE, standard error (SE), summary of the model selection criteria and showing the −*ℓ*, AIC, CAIC, BIC, W*, AD*, KS and P-value statistics in each model, for the four data sets respectively. In addition, the plots of the estimated pdf and cdf with empirical data in Figs 3–6 show closeness, confirming that MOW-E and MOW-W have a better fit for all data sets compared to other competing distributions.

**Table 7:** MLE and SE of the model parameters for data set I.

Model	MLE and (SE)			
MOW-E	0.0084	6.2384	0.3331	0.0487
$(\hat{\alpha }$,$\hat{c}$,$\hat{\beta }$,$\hat{\lambda }$)	(0.0102)	(0.8602)	(0.4434)	(0.0637)
MO-E	36.7180	1.2089		
$(\hat{\alpha }$,$\hat{\lambda }$)	(15.2164)	(0.1356)		
MO-W	0.1732326	4.2783684	4.7221390	
$(\hat{\alpha }$,$\hat{c}$,$\hat{\beta }$)	(0.1179)	(0.6901)	(0.5084)	
Weibull-E	3.3153	15.9402	4.3817	
($\hat{c}$,$\hat{\beta }$,$\hat{\lambda }$)	(0.3789761)	(1.0025)	(0.1452)	
Weibull-W	0.7337	2.1786	4.3658	3.0146
($\hat{c}$,$\hat{\beta }$,$\hat{\gamma }$,$\hat{\delta }$)	(0.0857)	(0.5183)	(0.1921	(0.0026)

**Table 8:** Model selection criteria for data set I.

Model	−*ℓ*	AIC	CAIC	BIC	W*	AD*	KS	p-value
MOW-E	47.055	102.110	105.277	108.444	0.0539	0.3393	0.099	0.868
MO-E	53.098	110.196	111.780	113.363	0.4262	2.2967	0.211	0.079
MO-W	49.814	105.629	108.004	110.379	0.2083	1.1220	0.161	0.31
Weibull-E	51.474	108.948	111.323	113.698	0.1980	1.1424	0.150	0.390
Weibull-W	51.256	110.514	113.681	116.848	0.2035	1.1932	0.136	0.515

**Table 9:** MLE and SE of the model parameters for data set II.

Model	MLE and (SE)			
MOW-E	0.1913	1.2272	3.7838	0.0173
$(\hat{\alpha }$,$\hat{c}$,$\hat{\beta }$,$\hat{\lambda }$)	(0.1118)	(0.1127)	(3.7369)	(0.0165)
MO-E	2.0907	0.0163		
$(\hat{\alpha }$,$\hat{\lambda }$)	(0.9718)	(0.0029)		
MO-W	5.2047	0.5829	20.3798	
$(\hat{\alpha }$,$\hat{c}$,$\hat{\beta }$,)	(7.8332)	(0.2379)	(32.9191)	
Weibull-E	0.9117	3.5951	0.0405	
$(\hat{c}$,$\hat{\beta }$,$\hat{\lambda }$)	(0.0506)	(8.3994)	(0.0938)	
Weibull-W	4.6322	1.8303	0.1966	4.1039
$(\hat{c}$,$\hat{\beta }$,$\hat{\gamma }$,$\hat{\delta }$)	(3.2733)	(0.3770)	(0.1393)	(2.6784)
MOEW	0.9615	0.8230	0.0106	
$(\hat{\alpha }$,$\hat{c}$,$\hat{\beta }$)	(0.0365)	(0.3903)	(0.0035)	

**Table 10:** Model selection criteria for data set II.

Model	−*ℓ*	AIC	CAIC	BIC	W*	AD*	KS	p-value
MOW-E	1032.821	2073.642	2080.115	2086.587	0.039	0.301	0.045	0.833
MO-E	1053.74	2111.479	2114.715	2117.952	1.027	6.562	0.128	0.004
MO-W	1042.078	2090.155	2095.01	2099.865	0.161	1.471	0.072	0.283
Weibull-E	1036.757	2079.514	2084.369	2089.224	0.160	0.996	0.060	0.503
Weibull-W	1036.762	2081.523	2087.996	2094.469	0.164	1.008	0.061	0.488
MOEW	1037.073	2080.145	2085	2089.855	0.4905	2.323	0.094	0.069

**Table 11:** MLE and SE of the model parameters for data set III.

Model	MLE and (SE)			
MOW-E	0.0367	8.0621	0.1863	0.0411
$(\hat{\alpha }$,$\hat{c}$,$\hat{\beta }$,$\hat{\lambda }$)	(0.0518)	(1.0050)	(0.1560)	(0.0330)
MO-E	27.6589	1.2273		
$(\hat{\alpha }$,$\hat{\lambda }$)	(7.4415)	(0.0980)		
MO-W	31.3285	2.1262	1.6883	
$(\hat{\alpha }$,$\hat{c}$,$\hat{\beta }$)	(28.6707)	(0.4824)	(0.4273)	
Weibull-E	5.0454	12.3338	3.7198	
$(\hat{c}$,$\hat{\beta }$,$\hat{\lambda }$)	(0.4557)	(70.2159)	(21.1685)	
Weibull-W	3.8592	1.3212	1.3033	2.6755
$(\hat{c}$,$\hat{\beta }$,$\hat{\gamma }$,$\hat{\delta }$)	(4.4738)	(2.4814)	(1.5116)	(4.0872)
MOEW	4.4823	1.0436	0.0047	
$(\hat{\alpha }$,$\hat{c}$,$\hat{\beta }$)	(0.2353)	(0.4158)	(0.0016)	

**Table 12:** Model selection criteria for data set III.

Model	−*ℓ*	AIC	CAIC	BIC	W*	AD*	KS	p-value
MOW-E	57.504	123.009	127.2955	131.581	0.067	0.410	0.086	0.734
MO-E	83.417	170.835	172.978	175.122	1.492	7.960	0.303	1.8e-05
MO-W	62.302	130.604	133.819	137.033	0.091	0.867	0.101	0.534
Weibull-E	61.957	129.910	130.317	136.339	0.125	0.937	0.087	0.719
Weibull-W	61.958	131.916	136.202	140.486	0.122	0.929	0.088	0.704
MOEW	62.763	131.527	134.742	137.957	0.157	1.242	0.115	0.372

**Table 13:** MLE and SE of the model parameters for data set IV.

Model	MLE and (SE)				
MOW-W	13.7716	0.6547	1.0645	5.1070	1.1438
$(\hat{\alpha }$,$\hat{c}$,$\hat{\beta }$,$\hat{\gamma }$,$\hat{\delta }$)	(16.4430)	(0.1879)	(1.1491)	(0.0987)	(0.0601)
MO-E	45.2295	2.7174			
$(\hat{\alpha }$,$\hat{\lambda }$)	(13.705)	(0.211)			
Weibull-E	5.7842	26.5451	16.2636		
$(\hat{c}$,$\hat{\beta }$,$\hat{\lambda }$)	(0.5722)	(0.6067)	(0.0235)		
Weibull-W	0.8052	4.0655	7.0696	1.3470	
$(\hat{c}$,$\hat{\beta }$,$\hat{\gamma }$,$\hat{\delta }$)	(0.0830)	(0.7357)	(0.3091)	(0.0025)	
Exp-E	30.1085	2.5837			
($\hat{\delta }$,$\hat{\lambda }$)	(8.9653)	(0.2332)			
Exp-W	11.8863	0.3347	0.5336		
($\hat{\alpha }$,$\hat{c}$,$\hat{\beta }$)	(5.5123)	(0.2395)	(0.0401)		

**Table 14:** Model selection criteria for data set IV.

Model	−*ℓ*	AIC	CAIC	BIC	W*	AD*	KS	p-value
MOW-W	12.045	34.089	39.447	44.805	0.084	0.568	0.101	0.536
MO-E	35.371	74.742	76.885	79.028	1.329	6.884	0.262	0.0003
Weibull-E	15.171	36.342	39.556	42.771	0.203	1.216	0.147	0.129
Weibull-W	15.043	38.086	42.372	46.658	0.196	1.223	0.139	0.177
Exp-E	31.392	66.784	68.927	71.071	0.805	4.361	0.229	0.003
Exp-W	17.150	40.301	43.515	46.730	0.276	1.571	0.148	0.122

The results in Tables 8–14 along with the plots in Figs 3–6 indicate the potentiality of the proposed distributions when comparing with competitive distributions using the selected data.

## 8 Regression modeling

Practically speaking, lifetimes are usually affected by some explanatory variables. Parametric models can be used to estimate univariate survival functions as well as can be solved regression problems. In this section, we present a new location regression model based on the MOW-W distribution.

### 8.1 The log MOW-W distribution

The regression model can be obtained by considering the log MOW-W (LMOW-W) distribution in which the location parameter depends on some explanatory variables ***v***. Re-defining some parameters of the random variable *X* which have the MOW-W in Eq (8), as $\gamma =\frac{1}{\sigma }$ and *δ* = *e*^*μ*^, then, the random variable *Y* = *log*(*X*) will have the LMOW-W distribution with the following pdf and survival function respectively

$$\begin{array}{c} f\left(y;\alpha ,c,\beta ,\sigma ,\mu \right)=\frac{\frac{\alpha c}{\sigma \beta^{c}}e^{c(\frac{y-\mu }{\sigma })}e^{-{\left(\frac{e^{\frac{y-\mu }{\sigma }}}{\beta }\right)}^{c}}}{{\left[1-\bar{\alpha }e^{-{\left(\frac{e^{\frac{y-\mu }{\sigma }}}{\beta }\right)}^{c}}\right]}^{2}}, \end{array}$$ (27)

and

$$\begin{array}{c} S\left(y;\alpha ,c,\beta ,\sigma ,\mu \right)=\frac{\alpha e^{-{\left(\frac{e^{\frac{y-\mu }{\sigma }}}{\beta }\right)}^{c}}}{1-\bar{\alpha }e^{-{\left(\frac{e^{\frac{y-\mu }{\sigma }}}{\beta }\right)}^{c}}}, \end{array}$$ (28)

where *α*, *c*, *β* are defined in Eq (8), *σ* > 0 is the scale parameter and μ∈R is a location parameter.

For the standardized random variable $Z=\frac{Y-\mu }{\sigma }$, its pdf can be derived as

$$\begin{array}{c} f\left(z;\alpha ,c,\beta \right)=\frac{\frac{\alpha c}{\sigma \beta^{c}}e^{cz}e^{-{\left(\frac{e^{z}}{\beta }\right)}^{c}}}{{\left[1-\bar{\alpha }e^{-{\left(\frac{e^{z}}{\beta }\right)}^{c}}\right]}^{2}}. \end{array}$$ (29)

### 8.2 The LMOW-W regression model for censored data

Assuming the survival time *X*_*i*_ of the *i*^*th*^ individual in the sample, for *i* = 1, …, *n*, and considering a set of *p* covariates such that **v**_*i*_ = (1, *v*_*i*1_, *v*_*i*2_, …, *v*_*ip*_)^*T*^, where the 1 is for the intercept term, the location-scale regression model relating to the response variable *y*_*i*_ = *log*(*x*_*i*_) with the explanatory variable vector **v** can be mathematically described from the LMOW-W as follows

$$\begin{array}{c} y_{i}={\text{v}}_{i}^{T}\tau +\sigma z_{i},\;i=1,\ldots ,n, \end{array}$$ (30)

where the random error *z*_*i*_ has density function in Eq (29) with unknown parameters *σ*, *α*, *c*, *β* > 0 and ***τ*** = (*τ*_0_, *τ*_1_, *τ*_2_, …, *τ*_*p*_)^*T*^ are the unknown regression coefficients of the *p* explanatory variables. To illustrate, *y*_*i*_ has the location parameter $\mu_{i}=v_{i}^{T}\tau$, in which the vector ***μ*** = (*μ*_1_, *μ*_2_, …, *μ*_*n*_)^*T*^ of the location parameter is described by the linear model ***μ*** = ***V_τ_***, such that ***V*** = (**v**_1_, **v**_2_, …, **v**_*n*_)^*T*^ is a known model matrix.

For an independent sample of *n* observations (*y*_1_ **v**_1_), …, (*y*_*n*_, **v**_*n*_), assume that *F* and *C* be the sets of individuals for which the response variable *y*_*i*_ is the log-lifetime and log-censoring, respectively. Considering the non-informative censoring such that the observed lifetimes and censoring times are independent, the random response is determined by *y*_*i*_ = {*log*(*x*_*i*_), *log*(*c*_*i*_)}. Then, the log-likelihood function for the vector of parameters ***ζ*** = (*α*, *c*, *β*, *σ*, ***τ***^*T*^)^*T*^ from model (30) can be obtained from

$$\begin{array}{c} \ell \left(\zeta \right)=\sum_{i\in F}^{}log\left[f\left(y_{i}\right)\right]+\sum_{i\in C}^{}log\left[S\left(y_{i}\right)\right], \end{array}$$

where *f*(*y*_*i*_) is the density function in Eq (27) and *S*(*y*_*i*_) is the survival function in Eq (28) of *Y*_*i*_. Thus, the log-likelihood function for ***ζ*** defined as

$$\begin{array}{c} \begin{array}{cc} \ell (\zeta )= & rlog(\frac{\alpha c}{\sigma \beta^{c}})+c\sum_{i\in F}^{}z_{i}-\frac{1}{\beta^{c}}\sum_{i\in F}^{}e^{cz_{i}}-2\sum_{i\in F}^{}log(1-\bar{\alpha }e^{-{\left(\frac{e^{z_{i}}}{\beta }\right)}^{c}})\\ & +\left(n-r\right)log\left(\alpha \right)-\frac{1}{\beta^{c}}\sum_{i\in C}^{}e^{cz_{i}}-\sum_{i\in C}^{}log(1-\bar{\alpha }e^{-{\left(\frac{e^{z_{i}}}{\beta }\right)}^{c}}), \end{array} \end{array}$$ (31)

where *r* is the number of uncensored observations (failures) and $Z_{i}=\frac{y_{i}-\mu_{i}}{\sigma }$, for $\mu_{i}=v_{i}^{T}\tau$.

The log-likelihood function in Eq (31) is then, maximized to find the MLEs of ***ζ***. For this case, numerical non-linear optimization procedures are commonly applied. The **optim** function in R can be used in order to find the MLEs.

### 8.3 Application for log Marshall-Olkin Weibull-Weibull regression model for censored data

The LMOW-W regression model is applied to fit data from an earlier study by [44], which compared treatment with radiotherapy only (Arm A) and radiotherapy plus chemotherapy (Arm B), for patients with head and neck cancer. The study included the survival time of 51 patients in arm A and 45 patients in arm B. Particularly, 9 patients in arm A and 14 patients in arm B failed to follow up and were considered censored. Only one predictor is considered in this study; *v*_1_ which represents the two-arms (Arm A = 0, Arm B = 1). Therefore, the LMOW-W regression model is considered as

$$\begin{array}{c} y_{i}=\tau_{0}+\tau_{1}{\text{v}}_{i1}+\sigma z_{i},\;i=1,\ldots ,96, \end{array}$$

where *z*_1_, …, *z*_96_ are independent random variables with pdf in Eq 29.

The results from LMOW-W model are compared with some alternative regression models, namely; the log-Weibull (LW) in [45], the log-gamma-Weibull (LGW) in [46], the Kumaraswamy-log-logistic (KumL) [47], the log-modified Weibull (LMW) in [48], the log-generalized modified Weibull (LGMW) in [49] and the log-Pareto Weibull generalized lambda (LPWGL) in [50].

Results in Table 15 suggest that the LMOW-W model has the best fit to the data set as it has the lowest AIC and BIC values.

**Table 15:** MLE and SE of the model parameters for regression data.

Model	MLE and (SE)						AIC	BIC
LMOW-W	*α* = 0.0025	*c* = 6.8453	*β* = 3.3778	*σ* = 5.1362	*τ*_0_ = 3.7406	*τ*_1_ = 0.5729	300.2	315.6
	(0.0004)	(4.0844)	(0.4705)	(2.8910)	(5.9183)	(0.5729)		
LW	-	-	-	*σ* = 1.1800	*τ*_0_ = 6.7873	*τ*_1_ = −0.7490	312.7	320.3
				(0.1082)	(0.2088)	(0.2772)		
LGW	-	-	*a* = 0.0264	*σ* = 0.0510	*τ*_0_ = 8.0113	*τ*_1_ = −0.6274	339.7	349.9
			(0.0079)	(0.0152)	(0.1458)	(0.1608)		
KumL	-	*a* = 16.2819	*b* = 312.91	*σ* = 5.8106	*τ*_0_ = 1.6912	*τ*_1_ = −0.6834	308.0	320.8
		(2.6189)	(24.86)	(0.3258)	(6.0235)	(0.2792)		
LMW	-	-	*α*_1_ = 1*e* − 8	*σ* = 1.1787	*τ*_0_ = 6.7841	*τ*_1_ = −0.7470	314.6	324.8
			(0.0000)	(0.1078)	(0.2081)	(0.2766)		
LGMW	-	*φ* = 1.6609	λ = 1*e* − 8	*σ* = 1.6404	*τ*_0_ = 6.4261	*τ*_1_ = −0.8841	313.4	326.2
		(0.8248)	(0.0000)	(0.4048)	(0.7432)	(0.3256)		
LPWGL	*β* = 12.2901	*s* = 279.1100	*b* = 0.0410	*τ* = 1.204	*τ*_0_ = 6.0071	*τ*_1_ = 0.7552	316.2	331.6
	(2.1498)	(48.2961)	(0.0051)	(0.1104)	(0.1232)	(0.2789)		

## 9 Conclusion

Weibull-G family introduced in [25] has advantage of adding more flexibility in shapes of a base line distribution and can be generalized in some other forms. This paper presented and studied the MOW-G family which combines both MO transformation with the Weibull-G to produce compound distributions with better performance. Specifically, two sub-models namely, MOW-E distribution and MOW-W distribution are presented. Some of the statistical properties of the proposed family are studied. Some of the properties related to moments, moment generating function, incomplete moments, Quantile, median, Renyi entropy, and distribution of order statistics are presented. To estimate the parameters of the model, the method of maximum likelihood is applied. Additionally, the performance of the MLEs of the two selected members is assessed via Monte Carlo simulation studies based on two criteria; bias and RMSE. The study exhibits a good performance when estimating the parameters of the proposed family using the maximum likelihood method. Four real data sets are fitted by the proposed distributions as well as compared with some selected competitive distributions. The results have been compared based on some efficiency measurements. These results demonstrate that both MOW-E and MOW-W outperform other distributions in terms of goodness-of-fit. Furthermore, we suggested the LMOW-W regression model, which provides more versatility, as demonstrated by a real-world application. Hence, it can be concluded that the newly proposed family of distributions have a wider range of applications in several disciplines.

## Supporting information

S1 Appendix (PDF)

S1 Text (TXT)
