# Correction: A new generalized family of distributions based on combining Marshal-Olkin transformation with T-X family

**DOI:** 10.1371/journal.pone.0293100

**Published:** 2023-10-12

**Authors:** Hadeel Klakattawi, Dawlah Alsulami, Mervat Abd Elaal, Sanku Dey, Lamya Baharith

There are errors in the Funding section. The correct Funding statement is: This project was funded by the Deanship of Scientific Research (DSR) at King Abdulaziz University, Jeddah, under grant no. G: 1467-247-1440. The authors, therefore, acknowledge with thanks DSR for technical and financial support. The funders had no role in study design, data collection and analysis, decision to publish, or preparation of the manuscript.
