# Supplementary material for: A new generalized family of distributions based on combining Marshal-Olkin transformation with T-X family
Source: PLoS One. 2022 Feb 9;17(2):e0263673. doi: 10.1371/journal.pone.0263673 (PMC8827489; doi:10.1371/journal.pone.0263673)
Supplement: S1 Appendix — (PDF) [file pone.0263673.s001.pdf]

Appendix

The cdf and pdf for some sub-models of MOW-G family are given in the following Table:

The cdf and pdf for some sub-models of MOW-G family

$G(x;\boldsymbol{\tau})$	cdf	pdf
Uniform$(x;0,r)$	$\dfrac{1-e^{-\left(-\frac{\log\left(1-\frac{x}{r}\right)}{\beta}\right)^{c}}}{1-\bar{\alpha}e^{-\left(\frac{-\log\left(1-\frac{x}{r}\right)}{\beta}\right)^{c}}}$,	$\dfrac{\frac{\alpha c}{\beta}\left(\frac{\frac{1}{r}}{1-\frac{x}{r}}\right)\left(\frac{-\log(1-\frac{x}{r})}{\beta}\right)^{c-1}e^{-\left(-\frac{\log\left(1-\frac{x}{r}\right)}{\beta}\right)^{c}}}{\left[1-\bar{\alpha}e^{-\left(-\frac{\log\left(1-\frac{x}{r}\right)}{\beta}\right)^{c}}\right]^{2}}$.
Rayliegh$(x;s)$	$\dfrac{1-e^{-\left(\frac{x^2}{\beta s^2}\right)^{c}}}{1-\bar{\alpha}e^{-\left(\frac{x^2}{\beta s^2}\right)^{c}}}$,	$\dfrac{\left(\frac{2\alpha c}{\beta^{c}s^{2c}}\right)x^{2c-1}e^{-\left(\frac{x^2}{\beta s^2}\right)^{c}}}{\left[1-\bar{\alpha}e^{-\left(\frac{x^2}{\beta s^2}\right)^{c}}\right]^{2}}$.
Chi-square $(x;r)$	$\dfrac{1-e^{-\left(\frac{-\log\left(1-\frac{\Gamma\left(\frac{x}{2},\frac{r}{2}\right)}{\Gamma\left(\frac{r}{2}\right)}\right)}{\beta}\right)^{c}}}{1-\bar{\alpha}e^{-\left(\frac{-\log\left(1-\frac{\Gamma\left(\frac{x}{2},\frac{r}{2}\right)}{\Gamma\left(\frac{r}{2}\right)}\right)}{\beta}\right)^{c}}}$,	$\dfrac{\frac{\alpha c}{\beta^{c}}\left(\frac{\frac{2^{-\frac{r}{2}}}{\Gamma\left(\frac{r}{2}\right)}x^{\frac{r}{2}-1}e^{-\frac{x}{2}}}{1-\frac{\Gamma\left(\frac{x}{2},\frac{r}{2}\right)}{\Gamma\left(\frac{r}{2}\right)}}\right)\left(-\log\left(1-\frac{\Gamma\left(\frac{x}{2},\frac{r}{2}\right)}{\Gamma\left(\frac{r}{2}\right)}\right)\right)^{c-1}e^{-\left(-\frac{\log\left(1-\frac{\Gamma\left(\frac{x}{2},\frac{r}{2}\right)}{\Gamma\left(\frac{r}{2}\right)}\right)}{\beta}\right)^{c}}}{\left[1-\bar{\alpha}e^{-\left(-\frac{\log\left(1-\frac{\Gamma\left(\frac{x}{2},\frac{r}{2}\right)}{\Gamma\left(\frac{r}{2}\right)}\right)}{\beta}\right)^{c}}\right]^{2}}$.
Gompertz$(x;r,s)$	$\dfrac{1-e^{-\left(\frac{s\left(e^{rx}-1\right)}{r\beta}\right)^{c}}}{1-\bar{\alpha}e^{-\left(\frac{s\left(e^{rx}-1\right)}{r\beta}\right)^{c}}}$,	$\dfrac{\frac{\alpha c}{\beta^{c}}se^{(rx)}\left(\frac{s}{r}\left(e^{rx}-1\right)\right)^{c-1}e^{-\left(\frac{s\left(e^{rx}-1\right)}{r\beta}\right)^{c}}}{\left[1-\bar{\alpha}e^{-\left(\frac{s(e^{rx}-1)}{r\beta}\right)^{c}}\right]^{2}}$.
Chen$(x;r,s)$	$\dfrac{1-e^{-\left(\frac{s\left(e^{x^{r}}-1\right)}{\beta}\right)^{c}}}{1-\bar{\alpha}e^{-\left(\frac{s\left(e^{x^{r}}-1\right)}{\beta}\right)^{c}}}$,	$\dfrac{\frac{\alpha c}{\beta^{c}}\left(rsx^{r-1}e^{x^{r}}\right)\left(s(e^{x^{r}}-1)\right)^{c-1}e^{-\left(\frac{s\left(e^{x^{r}}-1\right)}{\beta}\right)^{c}}}{\left[1-e^{-\left(\frac{s(e^{x^{r}}-1)}{\beta}\right)^{c}}\right]^{2}}$.
